# Frequency of Drug Resistance Gene Amplification in Clinical *Leishmania* Strains

**DOI:** 10.1155/2010/819060

**Published:** 2010-07-12

**Authors:** C. Mary, F. Faraut, M. Deniau, J. Dereure, K. Aoun, S. Ranque, R. Piarroux

**Affiliations:** ^1^Laboratoire de Parasitologie-Mycologie, Hôpital de la Timone, 264 Rue Saint Pierre, 13385 Marseille cedex 5, France; ^2^UMR 956, UPVM, Hôpital Henri Mondor, 94010 Créteil, France; ^3^Laboratoire de Parasitologie-Mycologie 163, Rue Auguste Broussonet, 34090 Montpellier, France; ^4^Institut Pasteur de Tunis, Place Pasteur B.P. 74 Tunis Belvédère, Tunisie 1002, Tunisia

## Abstract

Experimental studies about *Leishmania* resistance to metal and antifolates have pointed out that gene amplification is one of the main mechanisms of drug detoxification. Amplified genes code for adenosine triphosphate-dependent transporters (multidrug resistance and P-glycoproteins P), enzymes involved in trypanothione pathway, particularly gamma glutamyl cysteine synthase, and others involved in folates metabolism, such as dihydrofolate reductase and pterine reductase. The aim of this study was to detect and quantify the amplification of these genes in clinical strains of visceral leishmaniasis agents: *Leishmania infantum, L. donovani*, and *L. archibaldi*. Relative quantification experiments by means of real-time polymerase chain reaction showed that multidrug resistance gene amplification is the more frequent event. For P-glycoproteins P and dihydrofolate reductase genes, level of amplification was comparable to the level observed after in vitro selection of resistant clones. Gene amplification is therefore a common phenomenon in wild strains concurring to *Leishmania* genomic plasticity. This finding, which corroborates results of experimental studies, supports a better understanding of metal resistance selection and spreading in endemic areas.

## 1. Introduction

Visceral leishmaniasis (VL) constitutes a public health problem in East Africa, Asia, Mediterranean basin, and Central and South America. It is estimated that about 500 000 new cases occur each year. In East Africa and India, VL cause large-scale and tenacious epidemics with high case-fatality rates [1]. In numerous countries that cannot afford the cost of liposomal amphotericin B, first line treatment still relies on sodium stibogluconate or N-methyl glucamine [2]. Cases refractory to antimony treatment have been described for a long time in humans [3] as well as in dogs [4]. During the last decades, incidence of resistance to antimony drugs in *Leishmania spp*. increased dramatically in some VL foci [5]. The mechanisms contributing to drug resistance *in vivo* are poorly understood. *In vitro* studies of strains selected for metal resistance showed that several independent pathways could concur to the resistance phenotype [6]. Among them, overexpression of energy dependent transporters seems to play a major role in resistance to antimonials (reviewed in [7]). Genes coding for “ATP binding cassette” (ABC) transporters [8] and multidrug resistance (MDR) genes [9] have been shown to be amplified as extrachromosomal elements in strains selected* in vitro* for resistance to heavy-metals. Some works showed that genes that concur to glutathione and trypanothione synthesis can also be amplified in resistant parasites [10]. Gene amplification has been also described in *Leishmania* strains selected for resistance to methotrexate [11]. Other molecules are involved in trivalent antimony or arsenite compounds transport like aquaglyceroporins whose down regulation provides resistance to trivalent antimony and mutation could affect specifically metal transport [12]. 

However, all these findings derived from studies of strains whose resistance was induced *in vitro* and data about natural conditions are lacking. The aim of the present study was therefore to evaluate both the frequency of gene amplification and the amplification level of genes involved in drug resistance among *Leishmania* clinical strains.

## 2. Materials and Methods

### 2.1. Leishmania  Strains

The study included 90 *Leishmania *strains isolated from French and Sudanese VL foci.

Sixty *L. infantum* strains were collected in France from 16 HIV positive patients presenting with autochthonous VL. When relapses occurred, we performed successive parasite isolations: for each patient, the number of isolates varied from two to seven and the duration of the survey was 10 to 1800 days (Table 1).

Twenty seven strains (fifteen *L. donovani*, six *L. archibaldi* and six *L. infantum*), were isolated from 27 Sudanese patients. Any of these patients were infected by HIV. Three other strains (two *L. donovani* and one *L. infantum*) were isolated from Sudanese post-Kala-Azar dermatitidis leishmaniasis (PKDL) patients (Table 2). 

All strains were identified, typed, and cryopreserved at the National Reference Centre for Leishmaniasis (Montpellier, France).

A *Leishmania infantum* strain, isolated from a dog in an area without any therapeutic pressure (MCAN/82/GR/MON497), was used as reference (calibrator) for the relative quantification experiments. *In vitro* sensitivity assays have confirmed the sensitivity of this strain to antimonials [13]. It was grown using the same conditions as the studied strains.

### 2.2. DNA Extraction

Parasites were grown in RPMI medium (containing 10% foetal calf serum, gentamycin, all from Sigma); cells were harvested by centrifugation, and washed twice with PBS before DNA extraction. DNA was purified from 5 10^6^ promastigotes by means of a QiaAmp DNA mini kit (Qiagen ref 51034) according to the manufacturer specifications. Elution was performed with 100 *μ*L of DNAse free water and the concentration was measured by spectrophotometry. DNA solutions were kept frozen (−40°C) until use.

### 2.3. Relative Quantifications of the Genes Under Study

We focused on the following genes: two ATP-dependant drug transporters, MDR 1 and P-glycoproteins P A (PGP A); one gene involved in glutathione and trypanothione synthesis, the G-glutamylcysteine synthetase (GSH1); two genes involved in the folate metabolism pathways, dihydrofolate reductase-thymidlate synthase (DHFR-TS) and pterine reductase (PTR1). 

We measured their amplification levels by means of real-time PCR, relatively to invariant genes. We used two single copy genes as reference genes: the DNA polymerase [14] and the ascorbate-dependant peroxidase [15]. Primers and probe targetting the DNA polymerase gene were chosen according to Bretagne et al. [16]. For the ascorbate-dependant peroxidase and the genes under study, specific sequences were extracted from Genbank (http://www.ncbi.nlm.nih.gov/) and primers and TaqMan probes were designed by means of Primer3 software (http://frodo.wi.mit.edu/primer3). Table 3 summarizes the sequences of all primers and probes used in this study.

All assays were duplex PCR, performed in duplicate experiments, on a Stratagene MX 4000 thermal cycler with a multiplex QPCR ready-to-use mix (Stratagene, ref 600549-51). Each assay was performed by using 0.1 ng of sample DNA as template. Thermal profile was 94°C for 10 minutes for polymerase activation, 94°C 30 s, 54°C 30 s, 45 cycles. All the samples results were compared to those of the reference strain by means of the delta (delta Ct) method; this method calculate, for each sample, the level of the genes of interest relatively to the invariants genes and then standardize the results by means of the calibrator [17]. 

### 2.4. Statistical Analysis

Statistical analysis was computed using the SAS Stat 9.1.3 (SAS, Cary, NC, USA) statistical software. Whether the occurrence of gene amplification was associated with *Leishmania* species was tested for each studied gene using the Pearson Chi-squared test. 

## 3. Results

### 3.1. Thresholds

For each gene studied, we need to estimate a threshold for amplification. In this aim, we determined the gene amplification levels of the sensitive reference strain from ten biological replicates. We considered this level as a sensitive reference value. The threshold defining significant gene amplification was fixed by calculating the mean value of the 10 replicates augmented of 3 standard deviations to avoid over estimation of gene amplification. Table 4 presents means, standard deviations, and thresholds for the genes of interest; all amplification thresholds were therefore lower than 2, indicating that our assays were able to discriminate at least a two fold gene amplification relatively to the chosen reference.

### 3.2. Evidence of Drug Resistance Genes Amplification in Field Strains

Data concerning the intensity and the frequency of gene amplification in the strains under study are presented in Tables 1, 2, and 4. Statistics took only in account results of the initial strain isolated from the French patients.

MDR 1 gene was at least twice amplified in 30 of the 46 primitive strains, representing 65% of the cases. GSH was amplified in only three Sudanese strains (mean amplification level = 2.41). In the first strain, GSH amplification was associated with PGP A, MDR 1, and PTR1 amplification; in the second, GSH amplification was associated with PGP A and MDR 1 amplification; in the third, GSH amplification was associated with MDR 1 amplification.

Five out of the 16 French patients primitive strains showed DHFR gene amplification; in four of these strains MDR 1 gene was also amplified.

### 3.3. Stability of Gene Amplification Level among Successive Strains from the Same Patient

**Figure 1:**
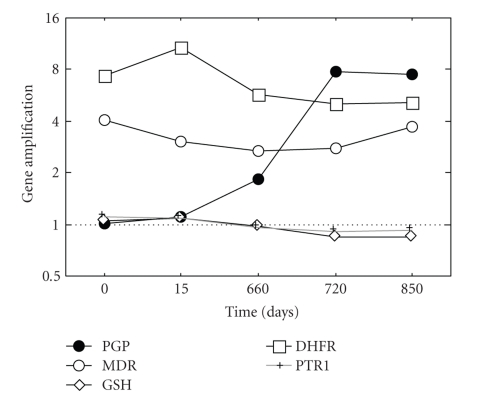
Variations of PGP A gene amplification among the successive Leishmania isolates from patient F8.

We analysed gene copy number variations in the sequential strains from the 16 French patients. Variations of the PGP A, MDR 1, GSH, DHFR and PTR genes amplification with respect to the initial level were less than or equal to 28%, 34%, 25%, 33%, and 10%, respectively, except for the increase of PGP A amplification level observed in one patient (F8). This case corresponded to a patient initially treated with N methyl glucamine and he relapsed 22 months after initial disease. For this patient, the two last strains, collected during relapse, presented with PGP A amplification levels of 7.84 and 7.49 instead of an initial level of 0.45 before the first treatment course (Figure 1).

### 3.4. Gene Amplification Frequency in Leishmania Species

We tested the eventual association between the species *L. archibaldi, L. donovani* (absent in the Mediterranean focus), *L. infantum* (present in the two studied foci), and gene amplification. Only MDR 1 amplification, which was observed in 83% of the *L. infantum* strains, was statistically (*P*  =  .04) associated with the species (Table 5). 

## 4. Discussion

Several studies aimed to document gene amplification among *Leishmania* genome of strains selected under *in vitro* drug pressure and its involvement in resistance mechanisms [18]. The first approach used hybridisation experiments after standardization of the samples by means of a housekeeping gene [8]. More recently, micro array technology was developed; a large number of genes could be analysed into a single experiment, improving the screening capacity. This technology confirmed the involvement of gene amplification in *Leishmania* resistance to antimony and was complemented by other techniques for finest quantification of the variable genes [10, 19]. A better accuracy is obtained by means of comparative real-time PCR, quantifying the number of copy of a target gene to one or more housekeeping genes. 

We analysed amplification of five genes involved in drug resistance on the basis of experimental studies. Our approach was reproducible enough to appreciate a two fold increase in gene amplification but presents some limiting aspects. A clear correlation between clinical response to therapy and gene amplification was impossible to establish because most of the French patients were treated with liposomal amphotericin B and we lack precise information about response to meglumine antimoniate administration in Sudanese cases. Other mechanisms than gene amplification are involved in resistance to treatment such as over expression, the two phenomenons being sometimes linked [20]. In this study, promastigotes were tested after 5 to 8 in vitro passages; we hypothesized that the studied characters were stable, as described by others authors *in vivo* [21] and *in vitro *[22]. Nevertheless, we believe our results highlight some significant aspects of the phenomenon of drug resistance of *Leishmania* that need further discussion.

Our main findings show that gene amplification, initially described in experimental studies about drug resistance of laboratory mutants, can also be encountered in *Leishmania* field strains.

The most frequently amplified gene was the MDR 1 gene, which was found amplified in 65% of the strains with an average amplification level of 6.98. The MDR 1 gene has been characterized in several *Leishmania* species. This gene was initially found to be amplified in mutants selected for vinblastine (V circles) [23] resistance and transfection experiments showed that the product of this gene can induce drug resistance by active extrusion of the drug. Experimental studies showed that efflux could be inhibited by verapamil [24]. This gene is highly homologous to the mammalian MDR genes involved in resistance to anticancer drugs. The amplification level of MDR 1 we observed in *Leishmania* was comparable to those reported for *Plasmodium falciparum* in experimental studies [25]. Resistance of *Plasmodium falciparum* to quinine has been associated with PfMDR1 over expression, was directly correlated to gene amplification, and confers cross resistance to other drugs [26]. The selection of MDR amplicons could be explained by the intensive, or perhaps exclusive, use of meglumine antimoniate for VL treatment in Sudan and in veterinary practice in France. So the frequency of MDR amplification may reflects the therapeutic pressure exerted on *Leishmania*. Katakura et al. [27] obtained stable transfectants for *L. amazonensis* MDR 1 gene at a copy number over 500 under selective pressure and, for PGP A, Guimond et al. [10] showed that a 2.2-fold increase detected by means of microarray technology was measured as a 12.7-fold increase by Southern blot. Our results showed that high copy number of putatively resistance genes can also be observed in natural conditions; one strain (S30) showed a very high copy number both for MDR 1 and PGP A, respectively, 63- and 22-fold. Performing duplex PCR kept the results clear of a technical error related to differences in sample amounts introduced in the reaction tubes.

The PGP A was found amplified in five strains with an average number of copy of 8.42, similar to the ratios reported by Guimond after selection of metal resistant strains [10]. PGP A belongs to the ATP binding cassette transporters family, a subfamily of multidrug resistance proteins (MRP). PGP A recognizes metal-thiol conjugates and confers resistance to trivalent metals (AS III or Sb III compounds) by accumulating the conjugates in intracellular vesicles close to the flagellar pocket [28]. The level of resistance conferred by transfection of this gene is dependent on the *Leishmania* species, suggesting that others factors concur to resistance. In another study, Haimeur found that the overproduction of PGP A was associated with GSH 1 gene amplification [8]. Our findings showed that such a concomitant amplification of these genes also occurs in field strains in 3 out of 5 cases of PGP A amplification. 

Legare showed that an increase of trypanothione synthesis concur to the detoxification of the reduced antimony compound [29]. GSH1 codes for the heavy subunit of gamma-glutamylcysteine synthase [30], one of the enzymes involved in trypanothione pathway and Grondin et al. showed that GSH1 is overexpressed consecutively to gene amplification in metal resistant strains [31]. In the strains under study, GSH amplification seems to be a minor event and further work has to be made for testing the hypothesis of an overexpression without gene amplification.

Amplification of the DHFR gene was only found in five French *L. infantum* strains. DHFR-TS is a bifunctional enzyme, involved in the main pathway of folates synthesis; a duplication of its genomic region was first described in methotrexate resistant mutants [32]. One can hypothesise that drug pressure was most important in the Mediterranean area, due to the extended use of trimethoprime in veterinary practice [33]. Guimond et al. found a 7.1 increase for DHFR gene amplification level in strains selected for resistance to methotrexate [10]. We show here that some natural strains presented with the same amplification magnitude (average = 10.6). DHFR amplification and mutations were also found in malaria parasites under natural or experimental drug pressure [34]. These mutations allowed to describe DHFR alleles involved in pyrimethamine resistance and to perform genetic studies about the spreading of resistance from Asia to Africa [35]. 

Amplification of the PTR1 gene appears uncommon in our collection of natural strains. This finding is consistent with the fact that antifolate chemotherapy is unsuccessful in trypanosomatids and that amplification of this gene would be a physiological consequence of inhibition of DHFR [36].

All these genes are known to be amplified as extrachromosomal elements. The R region for DHFR, the H region for PTR1 and PGP A were described after selection for resistance to methotrexate in *L. tarentolae* [37] and *L. major* [38]. In the present study, we do not observe such linkage, even in the F8 patient, showing the selection of amplified PGP A parasites. MDR genes are coded by V circles or D circles, depending on the drug used for their selection. LD1 region was the first extrachromosomal amplicon described in *Leishmania* and was present in about 15% of the wild-type strains [39]. These elements participate to the plasticity of the *Leishmania* genome. 

Except for MDR 1, statistics showed that gene amplification was not associated with *Leishmania* species in a significant manner. This might be explained by relative low number of strains displaying genes amplification in the study samples, leading to an insufficient power to detect such association.

Our findings illustrate the relevance of quantitative molecular tools for studying gene amplification potentially involved in *Leishmania* drug resistance. The high frequency and spreading of amplified genes among field strains is crucial for understanding drug resistance spreading and therapeutic failure in *Leishmania* species. However, these results, obtained from analysis of the promastigote stage, need to be confirmed on tissue amastigotes and clinical significance of such data warrants to be evaluated in further studies that should compare gene amplification to clinical outcome of antimony therapy. In addition, the fact that other mechanisms which could participate to antimony resistance in natural conditions, such as variable gene expression or altered proteins functionality, remains to be documented. 

## Figures and Tables

**Table 1:** Main characteristics of the isolates and results of relative quantification of resistance genes for French VL patients. NA: nonamplified.

Patients	Parasite identification	Site/time of isolation	PGP	MDR	GSH	DHFR	PTR1
F1	*L. infantum MON 24*	blood j 0	1.12	3.22	0.85	0.94	0.91
	*L. infantum MON 24*	blood j 30	0.78	2.83	0.79	0.67	0.96
	*L. infantum MON 24*	bone marrow j130	0.94	4.17	0.75	0.48	0.96

F2	*L. infantum MON 24*	blood j 0	3.07	6.1	0.93	1.04	1.05
	*L. infantum MON 24*	peau j10	3.64	4.86	0.88	0.97	0.91

F3	*L. infantum MON 33*	blood j 0	1.3	4.05	0.95	0.75	1.02
	*L. infantum MON 33*	blood j 90	1.67	3.87	0.94	0.66	1.03
	*L. infantum MON 33*	blood j 250	1.18	5.41	0.88	0.6	0.99
	*L. infantum MON 33*	blood j 540	0.94	4.83	0.86	0.81	0.98
	*L. infantum MON 33*	blood j 1020	1.6	4.4	0.78	0.75	0.86
	*L. infantum MON 33*	blood j 1440	1.32	3.67	0.73	0.83	0.84
	*L. infantum MON 33*	blood j 1830	1.01	4.82	0.73	0.73	0.93

F4	*L. infantum MON 1*	blood j 0	1.2	5.52	0.97	15.59	0.74
	*L. infantum MON 1*	blood j 100	1.21	6.26	1.01	16.2	0.73

F5	*L. infantum MON 1*	cavum j 0	1.78	3.01	1.01	1.48	1.05
	*L. infantum MON 1*	blood j 90	1.91	3.97	0.94	1.31	1.07
	*L. infantum MON 1*	blood j 180	1.57	3.44	0.71	0.87	1.11
	*L. infantum MON 1*	skin j 180	2.13	4.3	0.65	0.89	0.94

F6	*L. infantum MON 1*	blood j 0	1.53	12	1.25	11.5	1.11
	*L. infantum MON 1*	blood j 450	1.19	8.72	1.28	19.1	1.17
	*L. infantum MON 1*	blood j 810	1.29	8.4	1.64	12.8	1.12

F7	*L. infantum MON 24*	bone marrow j 0	1.12	3.94	0.86	0.71	0.85
	*L. infantum MON 24*	bone marrow j 30	1.13	3.97	0.83	0.62	0.77

F8	*L. infantum MON 1*	blood j 0	1.02	4.06	1.05	7.39	1.11
	*L. infantum MON 1*	bone marrow j 15	1.11	3.02	1.08	10.7	1.08
	*L. infantum MON 1*	blood j 660	1.82	2.68	0.97	5.71	0.96
	*L. infantum MON 1*	blood j 720	7.84	2.8	0.85	5.03	0.91
	*L. infantum MON 1*	blood j 850	7.49	3.73	0.85	5.09	0.93

F9	*L. infantum MON 24*	blood j 0	1.02	5.32	1	16.05	0.66
	*L. infantum MON 24*	blood j 10	0.88	4.61	0.8	16.8	0.67
	*L. infantum MON 24*	blood j 110	0.96	7.88	1.02	15.1	0.69
	*L. infantum MON 24*	blood j 390	0.94	7.4	0.67	9.98	0.64
	*L. infantum MON 24*	blood j 1140	0.98	8.67	1.42	5.89	0.66
	*L. infantum MON 24*	blood j 1240	0.95	6.68	1.3	11.7	0.68

F10	*L. infantum MON 24*	blood j 0	1.1	5.05	0.72	0.83	0.74
	*L. infantum MON 24*	blood j 15	0.62	4.01	0.58	0.81	0.71
	*L. infantum MON 24*	blood j 50	1.2	4.19	0.81	0.85	0.65
	*L. infantum MON 24*	blood j 80	1.13	3.67	0.65	0.96	0.67
	*L. infantum MON 24*	blood j 420	0.99	5.48	0.63	0.91	0.71

F11	*L. infantum MON 1*	bone marrow j 0	1.47	5.32	0.67	0.92	0.73
	*L. infantum MON 1*	bone marrow j 110	1.67	4.56	0.6	0.61	0.79

F12	*L. infantum MON 1*	bone marrow j 0	1.65	4.04	0.83	0.85	NA
	*L. infantum MON 1*	bone marrow j360	1.78	6.75	0.75	0.76	NA
	*L. infantum MON 1*	blood j 810	2.58	4.22	0.66	0.67	NA
	*L. infantum MON 1*	blood j 1800	2.07	4.58	0.85	0.67	NA

F13	*L. infantum MON 24*	bone marrow j 0	0.71	1.66	0.74	2.49	0.86
	*L. infantum MON 24*	skin j 4	1.28	2.17	0.81	1.19	0.81
	*L. infantum MON 24*	blood j 810	0.82	1.77	0.74	1.36	0.82
F14	*L. infantum MON 24*	blood j 0	0.76	5.22	0.87	0.69	0.8
	*L. infantum MON 24*	blood j 10	0.78	4.9	0.75	0.56	0.82
	*L. infantum MON 24*	blood j 165	0.95	4.89	0.61	0.73	0.84
	*L. infantum MON 24*	blood j 420	0.98	5.61	0.59	0.87	0.78
	*L. infantum MON 24*	blood j 630	0.66	4.56	0.63	1	0.75

F15	*L. infantum MON 24*	blood j 0	1	3.42	1.31	0.61	1
	*L. infantum MON 24*	blood j 240	0.89	3.97	0.69	0.73	0.93
	*L. infantum MON 24*	blood j 400	0.73	4.07	0.68	0.59	0.96
	*L. infantum MON 24*	blood j 580	1.48	5.09	0.64	0.8	0.95

F16	*L. infantum MON 1*	bone marrow j 0	0.86	3.68	0.82	1.53	0.91
	*L. infantum MON 1*	skin j 710	0.82	3.05	0.52	2.05	0.92
	*L. infantum MON 1*	blood j 1700	0.92	4.31	0.51	2.21	0.81

**Table 2:** Main characteristics of the isolates and results of relative quantification of resistance genes for Sudanese patients. All patients were visceral leishmaniasis patients except S28, S29, and S30 who presented with post kala-azar dermal leishmaniasis. NA: nonamplified.

Patients	Parasite identification	Site of isolation	PGP	MDR	GSH	DHFR	PTR1
S1	*L. donovani MON 18*	lymph node	0.98	1.21	0.85	1.01	1.03
S2	*L. donovani MON 18*	lymph node	4.47	19.4	2.93	0.93	1.49
S3	*L. donovani MON 18*	lymph node	1.28	2.71	0.92	0.98	0.85
S4	*L. donovani MON 18*	lymph node	0.78	1.21	1.04	1.03	2.03
S5	*L. donovani MON 18*	lymph node	0.97	6.16	1.61	1.02	1.03
S6	*L. donovani MON 18*	lymph node	0.8	5.49	0.62	0.75	0.86
S7	*L. donovani MON 18*	lymph node	0.82	NA	1.02	1.05	1.02
S8	*L. donovani MON 18*	lymph node	NA	0.96	0.85	NA	0.91
S9	*L. donovani MON 18*	lymph node	NA	0.78	0.95	NA	0.85
S10	*L. donovani MON 18*	lymph node	1.05	0.99	0.61	1.02	1.05
S11	*L. donovani MON 18*	lymph node	4.54	1.02	0.69	NA	NA
S12	*L. donovani MON 18*	lymph node	1.52	1.48	NA	1.07	0.98
s 13	*L. donovani MON 18*	lymph node	1.07	1.74	NA	NA	NA
S14	*L. donovani MON 18*	lymph node	1.17	2.72	1.04	0.98	2.7
S15	*L. donovani MON 18*	lymph node	0.94	4.68	0.95	0.95	0.86
S16	*L. archibaldi MON 257*	lymph node	0.87	0.98	1.04	1.02	NA
S17	*L. archibaldi MON 257*	lymph node	0.81	5.25	0.72	1.05	1.04
S18	*L. archibaldi MON 258*	lymph node	1.06	2.7	0.77	0.97	NA
S19	*L. archibaldi MON 257*	lymph node	0.92	1.33	0.98	0.85	0.83
S20	*L. archibaldi MON 257*	lymph node	1.13	4.47	0.79	1.03	NA
S21	*L. archibaldi MON 257*	lymph node	1.55	2.24	0.87	0.93	NA
S22	*L. infantum MON 30*	lymph node	7.83	3.62	1.12	0.83	0.97
S23	*L. infantum MON 30*	lymph node	0.99	1.74	0.77	0.92	NA
S24	*L. infantum MON 30*	lymph node	0.72	2.96	1.04	0.73	0.68
S25	*L. infantum MON 30*	lymph node	1.32	3.12	0.67	0.94	NA
S26	*L. infantum MON 30*	lymph node	1.04	1.03	1.01	1.02	NA
S27	*L. infantum MON 30*	lymph node	0.78	0.96	0.97	1.05	NA
S28	*L. infantum MON 267*	skin	0.93	7.13	2.48	0.95	NA
S29	*L. donovani MON 18*	skin	0.94	1.51	1.03	1.04	NA
S30	*L. donovani MON 18*	skin	22.2	62.8	1.83	0.63	1.1

**Table 3:** Primers and probes designed from the reference sequences of the studied genes.

	Forward primer	Reverse primer	Probe	Genbank access
PGP A	GAGGGTGTGCAGATGCGGTA	CATGAACGTCAGCAGCAGCG	FAM- CTTCCCACTCCCTGTCCGCCCGA- TAMRA	X17154
MDR 1	GCGACCTGAACCTGACGATC	GCCCGATCATCGACGACTTG	FAM-CGCACCCAGACGACCCAGAGAACG- TAMRA	L01572
GSH	CTCCTTTGCGACACCGATGT	GAGTCGTAGCGAGACTTGAGAAT	FAM-CGGCACCTCCTCCAAGCGGCG- TAMRA	Y10049
DHFR	GTACCTTGAGCTGATTGACC	AGATGTGAATGGGGTCCTTGTCC	FAM-CTGCTGACGACGAAGCGTGT- TAMRA	AY122331
PTR 1	GCTGTCTGTTTGCACTATCA	CTTGAAGAGGGTAACAGGTG	FAM-TTGGCAACGTTGCTCAGGTC- TAMRA	AY547305
DNA POL	TGTCGCTTGCAGACCAGATG	GCATCGCAGGTGTGAGCAC	HEX-CCAGGCTCGAAGTTGTTGCTGCCC- TAMRA	AF009141
ASC PEROX	ATGATCTCTGAAAAGCTGGA	AGGGATATCGAGACCTTTGT	HEX-GCTTCAAACCGGAATGCCTG- TAMRA	XM 001686044

**Table 4:** Reference values (means, standard deviations, thresholds) from the sensitive reference strain and amplification coefficients of the clinical primo-isolates displaying amplification ratios above the threshold values calculated from the reference strain.

		PGP A	MDR 1	GSH	DHFR	PTR1
Reference strain (calibrator, 10 replicates)	Mean value	1.06	1.00	1.07	1.06	1.02
	Standard deviation	0.27	0.33	0.22	0.27	0.11
	Threshold value	1.87	1.99	1.73	1.87	1.35

Isolates displaying amplified genes	Minimum value	3.07	2.24	1.83	2.49	1.49
	Maximum value	22.2	62.8	2.48	16.05	2.7
	Mean value	8.42	6.98	2.41	10.6	1.85
	Number of isolates	5	30	3	5	3

**Table 5:** Distribution of the isolates displaying amplified genes according to the Leishmania species. *Amplification observed only with *L. infantum*.

Species	PGP A	MDR 1	GSH	DHFR*	PTR
*N* (%) of amplified genes	44	45	44	42	33
*L. archibaldi* (*n* = 6)	0 (0.0)	4 (66.7)	0 (0.0)	0 (0.0)	0 (0.0)
*L. donovani *(*n* = 17)	3 (20.0)	7 (43.8)	2 (13.3)	0 (0.0)	3 (21.4)
*L. infantum *(*n* = 23)	2 (8.7)	19 (82.6)	1 (4.4)	5 (21.7)	0 (0.0)

Total	**5 (11.4)**	**30 (66.7)**	**3 (6.8)**	**5 (11.9)**	**3 (9.1)**
	*P* = .36	*P* = .04	*P* = .44	*P* = .10	*P* = .11
